# The role of vascular echography in the management of complications associated with central venous access for hemodialysis in cancer patients: two case reports and literature review

**DOI:** 10.1590/1677-5449.000418

**Published:** 2018

**Authors:** Fernanda Costa Sampaio Silva

**Affiliations:** 1 Hospital Aristides Maltez, Liga Bahiana Contra o Câncer, Unidade de Terapia Intensiva, Salvador, BA, Brasil.; 2 Centro Médico Hospital da Bahia, Departamento de Angiologia e Cirurgia Vascular, Salvador, BA, Brasil.

**Keywords:** central venous catheters, venous thrombosis, subclavian artery, ultrasonography, Doppler

## Abstract

Central venous catheter implantation for hemodialysis is commonly performed in large centers and its complications are sometimes associated with insufficient training of those who perform it, but may also be related to the patient’s clinical condition. The present study reports two cases of complications related to use of a short-stay catheter for hemodialysis. In the first case, the cannula was inadvertently inserted into the left subclavian artery, causing arterial thrombosis, which was conservatively managed and good collateral perfusion was documented with vascular echography. The second case illustrates an incidental finding of Central Venous Septic Thrombosis in a patient who had used a catheter for a week, which was treated with antibiotic therapy, anticoagulation, and ultrasound control. In both cases, surgical intervention would have been high risk because of the patients' poor prognosis. Vascular ultrasonography enabled monitoring of these clinical situations and use of less aggressive treatments.

## INTRODUCTION

 Central venous catheter (CVC) placement for hemodialysis is a common procedure at large centers, although the health professionals available have not always been properly trained to conduct it. 1 Complications that have been documented include inadvertent arterial puncture with a small needle (5%), passage of the cannula through the artery (0.1 to 0.8%) and consequent hemothorax, hematoma with obstruction of airways, pseudoaneurysm, arteriovenous fistula, and encephalic vascular accident. 2 These devices are associated with around 65% of cases of deep venous thrombosis of the upper limbs, 3 and the incidence of infection is around 1.1-7.5 per 1,000 catheters inserted in cancer patients. 4 The role of vascular ultrasonography in prevention, diagnosis, and ongoing management of these types of situations is illustrated in the cases reported below. 

## CASE DESCRIPTIONS

### Case 1

**Figure 1:**
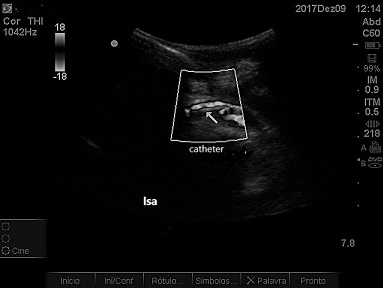
Catheter in the left subclavian artery (lsa).

**Figure 2:**
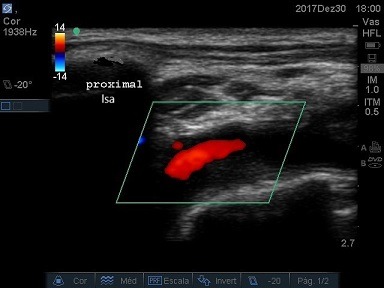
Left subclavian artery (lsa) after removal of the catheter: subacute luminal thrombus.

**Figure 3:**
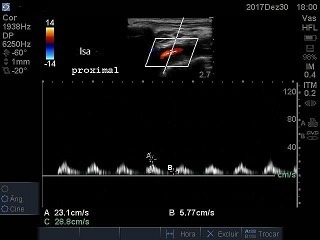
Left subclavian artery (lsa) after removal of the catheter: monophasic flow.

**Figure 4:**
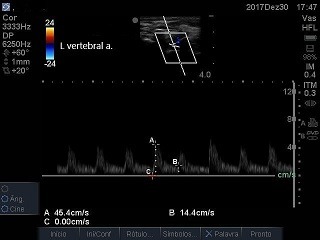
Left vertebral artery: normal flow.

**Figure 5:**
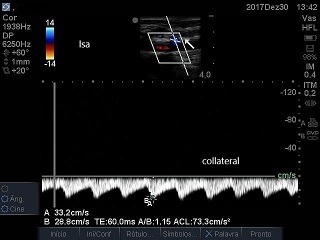
Reverse flow in branch of the left subclavian artery: dorsal subscapular artery.

**Figure 6:**
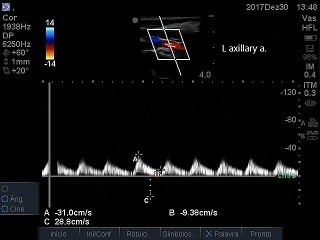
Axillary artery: slow, low-resistance flow.

 The patient was a 66-year-old male with advanced malignant prostate cancer, bone metastases, and kidney failure requiring dialysis. While an inpatient at a cancer hospital, he was transported to the intensive care unit (ICU) for catheter placement and a hemodialysis session. The professional on duty chose a left subclavian vein access, using anatomic landmarks. The blood aspirate at puncture appeared to be venous and the guidewire was advanced without difficulties, but after dilation of the tract and insertion of the catheter, retrograde pulsating flow was observed. Inadvertent positioning in the left subclavian artery (LSA) was confirmed by blood gas analysis and Doppler ultrasound ( Figure 1 ). The examination ruled out the possibility of injuries to the carotid or vertebral vessels, which had normal morphology and blood flow. Physical examination found 4+ brachial and radial pulses. The device was left in place and the patient was transferred to a hospital with vascular and endovascular surgery services. Inherent problems within the Brazilian National Health Service (SUS - Sistema Único de Saúde) delayed the transfer by 18 days. Since there was a risk of fatal complications, the catheter was not removed from the LSA and the patient was not given anticoagulation because of a recent history of melena. After transfer, the catheter was removed, but endovascular repair was not possible because a thrombus was seen in the arterial lumen. There was no bleeding or formation of hematoma, and left upper limb perfusion was maintained, although the brachial pulse was rated 2+ and the distal pulses were absent at that time. The patient was transferred back to the cancer hospital. Doppler vascular echography was conducted again, showing a subacute thrombus in the LSA, where flow was monophasic ( Figures 2 and 3 ), constituting subocclusion. The arterial thrombosis was in topography distal of the emergence of the vertebral artery, in which flow was laminar, anterograde and with velocities within the limits of normality ( Figure 4 ). At the subclavian-axillary transition, an arterial branch was observed with reversed flow that, based on topography, may have been the dorsal scapular artery ( Figure 5 ). The axillary ( Figure 6 ) and brachial arteries were patent and exhibited slow, low resistance flow, as did the radial and ulnar arteries. The conduct adopted in this case was watching and waiting since, in addition to the contraindication to anticoagulation already mentioned, the patient’s level of morbidity was elevated for an attempt at open revascularization and predictive indicators of the success of a possible bypass were unfavorable: the time elapsed since thrombus formation (22 days), the poor prognosis of the patient’s cancer, and the presence of kidney failure. The patient was observed for a further 2 weeks and did not show any sign of cyanosis, pain at rest, or trophic lesions. He was discharged from hospital for palliative home care. 

### Case 2

**Figure 7:**
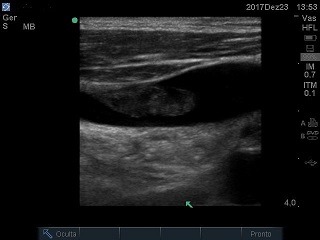
Free-floating thrombus in the right internal jugular vein.

**Figure 8:**
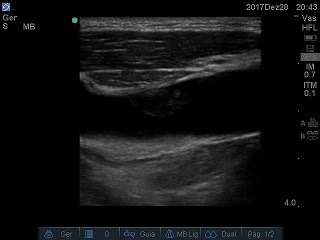
Progress of thrombus in the right internal jugular vein.

 The patient was a 56-year male who had been admitted to a cancer hospital because of malignant bladder cancer, with a history of radical surgical cystectomy and nephrostomies. He suffered kidney failure requiring dialysis and underwent CVC placement via the right internal jugular vein (RIJV) with no intercurrent conditions. While in hospital, treatment for pyelonephritis was initiated. Concurrently, the CVC became detached and vascular access was lost. Since nitrogen compounds were stabilized and there was urine production via the left nephrostomy, he did not need hemodialysis for 9 days, when his renal function deteriorated and he exhibited hyperkalemia. At this point he was transferred to the ICU for another CVC fitting. During the mapping process with vascular ultrasonography conducted to define the best puncture site, a heterogeneous thrombus with a free-floating tail was observed in the RIJV ( Figure 7 ), where the previous catheter had dwelt for 1 week. The contralateral internal jugular vein was patent and was chosen for the second vascular access, in view of the urgency of dialysis. Since he had no history of recent bleeding, the patient was treated according to the CHEST 2016 recommendations, 5 with full anticoagulation using unfractionated heparin and warfarin, withdrawing the heparin once his international normalized ratio (INR) reached the therapeutic range. He exhibited shivering after hemodialysis had been started via the catheter. Blood samples were taken for cultures and a wide spectrum antimicrobial regimen was initiated, since it was considered that cutaneous colonization by staphylococcus could have occurred, with a possibility of hematogenic dissemination of the urinary bacteria. One week later, the patient was no longer exhibiting fever or shivering. Another Doppler ultrasound examination was conducted, showing that the thrombosis was still present, but the images suggested that it had receded ( Figure 8 ) and were compatible with the organization phase of a thrombus. The patient remained under investigation for a suspected relapsed tumor in the right kidney and the recommendation was to maintain anticoagulation for 3 months. 

## DISCUSSION

 These two cases illustrate complications related to fitting a short-stay CVC for hemodialysis. Case 1 involved inadvertent placement of the access into the LSA, which is a rare complication, but can be fatal if managed incorrectly. Guilbert et al. documented greater morbidity and mortality when the conduct adopted is simple removal followed by manual compression of the site. 2 Although endovascular repair of traumatic lesions in the axillary-subclavian area is feasible in around 50% of cases, 6 the patient in this case had subacute thrombosis in the arterial bed, caused by the catheter being left in place for 18 days without anticoagulation. A study by Nicolajsen et al. 7 documented a 10.5% risk of myocardial infarction and 14.4% risk of encephalic vascular accident in cancer patients during the course of peripheral arterial thrombosis. There are also robust references to associations between cancer and reduced patency in vascular procedures conducted because of critical limb ischemia. 8 Vascular ultrasonography was of the utmost importance to documentation of the injury and of the circulatory status of the limb after the catheter had been removed. Since there was no bleeding from the insertion site and the limb was clinically compensated with good flow through collateral arteries, conservative management was the best choice. 

 Case 2 involved central venous septic thrombosis, which is a rare entity in its symptomatic form (0.8%). However, autopsy series report incidence rates in the range of 6.5-36.7%. 9 Guidelines published by the Brazilian Society of Angiology and Vascular Surgery 10 recommend treating CVC-related thrombosis with anticoagulation for 3 months using low molecular weight heparin or antivitamin K. The CHEST 2016 5 guidelines recommend anticoagulation rather than thrombolysis in cases of venous thrombosis of an upper limb or proximal veins, except if there is a high probability of progression to postthrombotic syndrome. There is no consensus on treatment of infected thrombi and there are scant up to date studies and few case reports in the literature. Hoffman and Greenfield 9 published a description of a case of subclavian-jugular central venous septic thrombosis treated using a superior vena cava filter, followed by jugular thromboembolectomy. The patient in case 2 had chronic renal failure and placement of a superior vena cava filter would have limited the possibilities for hemodialysis venous access. In 1986, Ang and Brown 11 published a series of seven cases of septic venous thrombosis and already argued in favor of less invasive treatment, with venous antibiotic therapy and full anticoagulation, reserving thrombectomy or resection of the venous segment involved for refractory cases. In case 2, vascular ultrasonography for pre-puncture venous mapping avoided iatrogenic embolization of the septic thrombus, in addition to proving a useful tool for documenting its progression. 

 While there is a lack of randomized studies comparing the technical safety of catheter insertion using anatomic landmarks with ultrasound-guided puncture, some studies suggest that the incidence of complications is lower when ultrasound is used during the procedure. In 2014, Zottele Bomfim et al. 12 published a study conducted at the Hospital A. C. Camargo, in São Paulo, Brazil, in which 100 cancer patients were divided into two groups and analyzed in terms of the rates of complications associated with placement of a valved versus a non-valved catheter for chemotherapy. While the objective of the study was to compare these two types of catheters, it is interesting to note the existence of a protocol for insertion of these devices, which ensured that all of the people studied had an ultrasonographic assessment of the jugular and subclavian veins prior to puncture, followed by ultrasound-guided CVC placement. In that study, no complications related to puncture were reported, and there were no catheter-related thrombotic events during follow-up of the cases. The authors argued that using ultrasound reduces the number of punctures needed, consequently reducing the chance of damage to vein walls, which could be related to the pathogenesis of thrombosis in these patients. 

 Additional studies are needed to determine the true role of vascular ultrasonography in prevention and management of complications associated with CVC placement . However, since echography is an accessible resource of relatively low cost and does not involve additional risks to patients, its routine use should be encouraged. 
