# Supplementary material for: The role of vascular echography in the management of complications associated with central venous access for hemodialysis in cancer patients: two case reports and literature review
Source: J Vasc Bras. 2018 Jul-Sep;17(3):257–61. doi: 10.1590/1677-5449.000418 (PMC6326130; doi:10.1590/1677-5449.000418)
Supplement: Translation

# Translation: O papel da ecografia vascular no manejo das complicações associadas ao acesso venoso central para hemodiálise em pacientes oncológicos: relato de dois casos e revisão da literatura

**Authors:** Fernanda Costa Sampaio Silva

**Affiliations:** 1 Hospital Aristides Maltez, Liga Bahiana Contra o Câncer, Unidade de Terapia Intensiva, Salvador, BA, Brasil.; 2 Centro Médico Hospital da Bahia, Departamento de Angiologia e Cirurgia Vascular, Salvador, BA, Brasil.

**Keywords:** cateteres venosos centrais, trombose venosa, artéria subclávia, ultrassonografia, Doppler

## Abstract

O implante de cateter venoso central para hemodiálise é comumente realizado em grandes centros e suas complicações estão, por vezes, associadas ao treinamento insuficiente de quem o realiza, mas também às condições clínicas do próprio doente. O presente estudo relata dois casos de intercorrências relacionadas ao uso do cateter de curta permanência para hemodiálise. No primeiro caso, houve inserção inadvertida da cânula na artéria subclávia esquerda e consequente trombose arterial, que foi conduzida conservadoramente, documentando-se uma boa perfusão colateral com ecografia vascular. O segundo caso ilustra o achado incidental de uma trombose venosa séptica central em paciente que havia feito uso do cateter por uma semana, optando-se por tratá-la com antibioticoterapia, anticoagulação plena e controle ecográfico. Em ambos os casos, a intervenção cirúrgica seria de alto risco devido ao prognóstico reservado dos doentes. A ultrassonografia vascular permitiu o monitoramento das situações clínicas e o emprego de terapêutica menos agressiva.

## INTRODUÇÃO

 O implante de cateter venoso central (CVC) para hemodiálise é um procedimento comum em grandes centros, embora nem sempre o profissional disponível seja devidamente treinado para realizá-lo 1 . Entre as complicações documentadas estão a punção arterial inadvertida com agulha pequena (5%), a passagem da cânula na artéria (0,1 a 0,8%) e consequente ocorrência de hemotórax, hematoma com obstrução de vias aéreas, pseudoaneurisma, fístula arteriovenosa e acidente vascular encefálico 2 . O dispositivo associa-se a cerca de 65% dos casos de trombose venosa profunda do membro superior 3 , e a incidência de infecção está entre 1,1-7,5 por 1.000 cateteres inseridos em pacientes oncológicos 4 . O papel da ultrassonografia vascular na prevenção, no diagnóstico e na condução evolutiva das referidas situações encontra-se ilustrado nos casos a seguir. 

## DESCRIÇÃO DO CASO

### Caso 1

 Paciente masculino, 66 anos, tinha neoplasia maligna avançada da próstata, com metástases ósseas e insuficiência renal dialítica. Internado em um hospital oncológico, foi encaminhado à unidade de terapia intensiva (UTI) para implante de cateter e sessão de hemodiálise. O plantonista optou pelo acesso em veia subclávia esquerda, utilizando marcadores anatômicos como guias. O sangue aspirado na punção parecia de natureza venosa e a passagem do fio-guia ocorreu sem dificuldades, mas, após a dilatação do pertuito e o implante do cateter, houve retorno de fluxo pulsátil. O posicionamento inadvertido na artéria subclávia esquerda (ASCE) foi confirmado por gasometria e Doppler ( Figura 1 ). O exame afastou a possibilidade de lesão em carótidas e vertebrais, cuja morfologia e fluxo eram normais. O exame físico demonstrou pulsos braquial e radial 4+. O dispositivo foi mantido *in situ*, e o doente foi encaminhado para um hospital com serviço de cirurgia vascular e endovascular. Dificuldades inerentes ao Sistema Único de Saúde brasileiro levaram à demora de 18 dias na transferência. Havendo risco de complicações fatais, o cateter não foi removido da ASCE e o paciente não foi anticoagulado devido à história recente de melena. Após a transferência, o cateter foi removido, mas o reparo endovascular não foi possível devido à visualização de trombo no lúmen arterial. Não houve sangramento ou formação de hematoma, e o membro superior esquerdo manteve-se perfundido, embora o pulso braquial fosse 2+ e os pulsos distais estivessem ausentes naquele momento. O paciente retornou ao hospital oncológico de origem. A ecografia vascular com Doppler foi realizada novamente e revelou presença de trombo subagudo em ASCE, onde o fluxo era monofásico ( Figuras 2 e 3 ), configurando padrão de suboclusão. A topografia da trombose arterial foi distal à emergência da artéria vertebral. Nesta última, o fluxo era laminar, anterógrado e com velocidades dentro do limite da normalidade ( Figura 4 ). Observou-se, na transição subclávia-axilar, a presença de ramo arterial com fluxo invertido, o qual, pela topografia, poderia corresponder à artéria escapular dorsal ( Figura 5 ). As artérias axilar ( Figura 6 ) e braquiais encontravam-se pérvias e tinham fluxo desacelerado de baixa resistência, assim como as artérias radial e ulnar. A conduta para o caso foi expectante, considerando-se, além da mencionada contraindicação para anticoagulação, a elevada morbidade para a revascularização aberta no doente em questão e os preditores desfavoráveis à patência de um possível *bypass*: tempo de evolução do trombo (22 dias), mau prognóstico da neoplasia e presença de insuficiência renal. O paciente foi observado por mais 2 semanas, não apresentando cianose, dor de repouso ou lesão trófica. Recebeu alta hospitalar para cuidados paliativos domiciliares. 

**Figura 1:** Cateter em artéria subclávia esquerda (asce).

**Figura 2:** Artéria subclávia esquerda (asce) após a remoção do cateter: trombo luminal subagudo.

**Figura 3:** Artéria subclávia esquerda após a remoção do cateter: fluxo monofásico.

**Figura 4:** Artéria vertebral esquerda: fluxo normal.

**Figura 5:** Fluxo invertido em ramo da artéria subclávia esquerda: artéria subescapular dorsal.

**Figura 6:** Artéria axilar: fluxo desacelerado de baixa resistência.

### Caso 2

 Paciente masculino, 56 anos, estava sendo acompanhado em hospital oncológico por neoplasia maligna da bexiga, antecedentes cirúrgicos de cistectomia radical e nefrostomias. Evoluiu com insuficiência renal dialítica e recebeu implante de CVC na veia jugular interna direita (VJID), sem intercorrências. Durante o internamento, iniciou tratamento para pielonefrite. Paralelamente, evoluiu com exteriorização e perda do acesso vascular. Tendo em vista a estabilização das escórias nitrogenadas e produção de urina pela nefrostomia esquerda, permaneceu sem necessidade de hemodiálise por 9 dias, quando apresentou piora da função renal e hipercalemia. Na ocasião, foi encaminhado à UTI para novo implante de CVC. Ao mapeamento com ultrassonografia vascular para definir o melhor sítio de punção, observou-se a presença de trombo heterogêneo com cauda flutuante em VJID ( Figura 7 ), sítio onde o cateter prévio permaneceu por 1 semana. A veia jugular interna contralateral encontrava-se pérvia, sendo eleita para o acesso vascular em questão, considerando-se a urgência dialítica. Não havendo história de sangramento recente, o paciente foi tratado conforme as recomendações do CHEST 2016 5 , com anticoagulação plena com heparina não fracionada, concomitante à varfarina, suspendendo-se a heparina após a razão normalizada internacional (RNI) atingir a faixa terapêutica. O paciente apresentou calafrios após iniciar a hemodiálise pelo novo cateter. Coletadas as hemoculturas, iniciou-se esquema antimicrobiano amplo, ponderando-se a colonização cutânea por estafilococos e a possibilidade de disseminação hematogênica do germe urinário. Decorrida uma semana, o paciente não apresentava mais febre ou calafrios. Um novo Doppler foi realizado, evidenciando ainda a presença da trombose, porém a imagem sugeria retração ( Figura 8 ), compatível com a fase de organização do trombo. O paciente prosseguiu em investigação para suspeita de recidiva tumoral em rim direito, com recomendação de manter a anticoagulação por 3 meses. 

**Figura 7:** Trombo flutuante em veia jugular interna direita.

**Figura 8:** Evolução do trombo em veia jugular interna direita.

## DISCUSSÃO

 Ambos os casos ilustram complicações relacionadas ao implante do CVC de curta permanência para hemodiálise. O caso 1 descreve a passagem inadvertida do acesso na ASCE, complicação rara, porém fatal quando não conduzida corretamente. Guilbert et al. documentaram uma maior morbimortalidade quando a conduta adotada é a simples remoção seguida de compressão manual do sítio 2 . Embora o reparo endovascular das lesões traumáticas no território axilo-subclávio seja factível em cerca de 50% dos casos 6 , o paciente em questão apresentou trombose subaguda no leito arterial, consequente à permanência, por 18 dias, do cateter *in situ* sem anticoagulação. O estudo de Nicolajsen et al. 7 demonstrou um risco de 10,5% para infarto do miocárdio e 14,4% para acidente vascular encefálico nos pacientes oncológicos em curso de trombose arterial periférica. Há também fortes referências à associação entre câncer e patência reduzida dos procedimentos vasculares para isquemia crítica dos membros 8 . A ultrassonografia vascular foi de suma importância na documentação da lesão e do estado circulatório do membro após a remoção do cateter. Não havendo sangramento pelo sítio de inserção, estando o membro compensado clinicamente por um bom fluxo arterial colateral, a conduta conservadora foi a mais adequada. 

 O caso 2 é um relato de trombose venosa séptica central, entidade rara (0,8%) na sua forma sintomática. Entretanto, séries de autópsias descrevem uma incidência entre 6,5-36,7% 9 . As diretrizes da Sociedade Brasileira de Angiologia e Cirurgia Vascular 10 orientam, para o tratamento da trombose relacionada a CVC, anticoagulação por 3 meses com heparina de baixo peso molecular ou antivitamina K. As diretrizes do CHEST 2016 5 recomendam anticoagulação em vez de trombólise nos casos de trombose venosa do membro superior ou veias proximais, exceto quando há elevada probabilidade para evolução com síndrome pós-trombótica. Para trombo infectado, não há consenso quanto ao tratamento e a literatura é pobre em estudos atuais, além de limitados a relatos de casos. Hoffman e Greenfield 9 publicaram um caso de trombose venosa séptica subclávia-jugular tratada com filtro de veia cava superior, seguido de tromboembolectomia jugular. O paciente do caso 2 era renal crônico e, nele, a presença do filtro de cava superior limitaria as possibilidades de acesso venoso para hemodiálise. Em 1986, Ang e Brown 11 publicaram uma série de sete casos de trombose venosa séptica e, naquela época, já defendiam um tratamento menos invasivo com antibioticoterapia venosa e anticoagulação plena, reservando-se a trombectomia ou ressecção do segmento venoso afetado para os casos refratários. No caso 2, a ultrassonografia vascular para mapeamento venoso pré-punção evitou a embolização iatrogênica do trombo séptico, além de constituir ferramenta útil na documentação de sua evolução. 

 Embora haja carência de estudos randomizados comparando a segurança técnica entre a inserção de cateteres direcionada por marcadores anatômicos e a punção ecoguiada, alguns trabalhos sugerem menor incidência de complicações quando o ultrassom é utilizado durante o procedimento. Em 2014, Zottele Bomfim et al. 12 publicaram um estudo realizado no Hospital A. C. Camargo, São Paulo, Brasil, no qual 100 pacientes oncológicos foram divididos em dois grupos e analisados quanto a taxas de complicações associadas ao implante de cateter valvulado *versus* não valvulado para quimioterapia. Conquanto o objetivo do trabalho fosse comparar os dois tipos de cateteres, é interessante notar a existência de um protocolo de inserção do dispositivo, no qual todos os indivíduos estudados tiveram avaliação ultrassonográfica das veias jugulares e subclávias precedendo a punção, seguida do implante ecoguiado do CVC. No referido estudo, nenhuma complicação relacionada à punção foi relatada, assim como também não foi documentado nenhum evento de trombose associado ao cateter no seguimento dos casos. Os autores argumentam que o uso do ultrassom reduz o número de punções necessárias, consequentemente reduzindo a chance de lesão à parede das veias, o que pode estar relacionado à patogênese da trombose nesses indivíduos. 

 Estudos adicionais são necessários para determinar o real papel da ultrassonografia vascular na prevenção e no manejo das complicações associadas ao implante de CVCs. Entretanto, sendo a ecografia um recurso acessível, de relativo baixo custo e que não agrega riscos adicionais ao paciente, seu uso rotineiro deve ser encorajado.
